# Comparison of tick-borne encephalitis between children and adults—analysis of 669 patients

**DOI:** 10.1007/s13365-020-00856-x

**Published:** 2020-06-10

**Authors:** Katarzyna Krawczuk, Piotr Czupryna, Sławomir Pancewicz, Elżbieta Ołdak, Anna Moniuszko-Malinowska

**Affiliations:** 1 Department of Pediatric Infectious Diseases, Medical University of Bialystok, Białystok, Poland; 2 Department of Infectious Diseases and Neuroinfections, Medical University in Bialystok, Żurawia 14, 15-540 Białystok, Poland

**Keywords:** TBE, Children, Adults

## Abstract

The aim of our study was to compare the course of TBE in children and adults. A retrospective analysis of the medical records of 669 patients was performed. The patients were categorized into 2 groups: Group I with 68 children and group II with 601 adults. TBE symptoms in children were milder compared with adults, with meningitis in 97% of cases. In adults, meningoencephalitis and meningoencephalomyelitis made up 49.26% of cases. Nausea and vomiting are more frequent in children, while neurological manifestations are more frequent in adults. There were no differences in CSF pleocytosis at the onset of disease in both groups, while CSF protein concentration was higher in adults. Children treated with corticosteroids over 7 days had higher checkup pleocytosis than pleocytosis at the onset of disease compared with adults. Corticosteroid use prolongs the disease duration but does not influence the development of TBE sequelae. Children had more favourable outcomes than adult patients.

## Introduction

Tick-borne encephalitis (TBE) is a common infectious disease in northeastern and central Europe that affects adults and children. TBE is the clinical presentation of infection with tick-borne encephalitis virus (TBEV), a member of the *Flavivirus* genus. There are 3 subtypes of TBEV: the European subtype (endemic in rural and forested areas of central, eastern and northern Europe), the Far-Eastern subtype (endemic in Far-Eastern Russia and in forested regions of China and Japan), and the Siberian subtype (endemic in the Urals region, Siberia and Far-Eastern Russia, and in some areas in northeastern Europe) (http://ecdc.europa.eu; Kartagina et al. 2013; Pancewicz et al. 2015).

The number of TBE cases in Europe varies depending on country and ranges from a few cases up to over 600 cases annually (Beauté et al. 2018). In Poland, the TBE notification rate increased in 1993 and has remained stable since then (Kaiser 2008). According to the data of the National Institute of Public Health-National Institute of Hygiene (the national centre for infectious diseases epidemiology), in Poland, the annual number of TBE cases fluctuates between 200 and 300. In 2004, 262 cases (0.62 per 100,000 inhabitants) were reported, and in 2018, 191 cases (0.51 per 100,000 inhabitants) were reported (Valarcher et al. 2015). The cases reported in Podlaskie Province accounted for 46% of all cases reported in our country.

TBE may take various clinical courses and forms, such as fever, meningitis, meningoencephalitis or meningoencephalomyelitis (Zajkowska et al. 2011; Steffen 2019). Meningitis is usually mild, and patients recover after 2–3 weeks of symptomatic treatment. In patients diagnosed with meningoencephalitis or meningoencephalomyelitis, the course of the disease is more severe. The overall case fatality rate is relatively low (ca. 2%) in European cases, and fatal outcomes are more often observed in elderly or immunodeficient patients but may also occur in young patients (Taba et al. 2017). It is known that the clinical course of TBE may vary depending on many factors, including the age of patients.

In the medical literature, few studies focus on differences in the course of diseases transmitted by ticks in children and adults (Arnez and Avsic-Zupanc 2009). This matter has not been studied in Poland.

## Aim

The aim of our study was to compare the epidemiological features, clinical course and laboratory findings of TBE in children and adults, residents of endemic areas of northeastern Poland.

## Material and methods

This was a retrospective analysis of the medical records of 669 patients with TBE hospitalized in 2004–2015. All patients hospitalized because of TBE in this period of time were included in the study. There was no special selection of patients performed.

The patients were categorized into 2 groups:

Group I included 68 children with TBE who were hospitalized in the Children’s Infectious Ward of the Provincial Hospital and later transferred to the University Hospital and in the Department of Paediatric Infectious Diseases, Medical University of Bialystok, Poland.

Group II included 601 adults with TBE who were hospitalized in the Department of Infectious Diseases and Neuroinfections, Medical University of Bialystok, Poland.

These infectious disease wards serve almost the entire population of the Podlaskie Province.

Medical data, such as patient age, sex, place of residence, history of a tick bite, subjective complaints, general examination results, neurological and psychiatric sequelae, laboratory parameters and treatment, were analysed.

In all cases, the diagnosis of TBE was confirmed by the detection of specific antibodies with enzyme-linked immunosorbent assay (ELISA) using the Virion/Serion kit (Wurzburg, Germany) according to the manufacturer’s instructions during hospitalization.

TBE-specific IgM and IgG antibody titers were measured using the Enzygnost Anti-TBE/FSME Virus Siemens test (enzyme immunoassay for the qualitative detection and quantitative determination of specific antibodies to the TBE virus in human serum and plasma). For the quantitative antibody determination of anti-TBE virus IgG, the measuring range was 7–700 U/ml.

The result of the IgM test was quantified by forming a quotient from the absorbance value of the test sample and the appropriate cut-off as the index. The index for the upper margin for retesting (“retest index”) was calculated by dividing the value of the upper margin for retesting (cut-off + 0.100) by the cut-off. An index 1.4-fold higher than the retest index was considered a positive result according to the instructions provided by the manufacturer.

Diagnosis of TBE was made based on the clinical presentation, presence of inflammatory parameters in the CSF and specific IgM and IgG antibodies in serum and CSF according to the case definition, i.e. the presence of clinical signs of meningitis, meningoencephalitis or meningoencephalomyelitis, an epidemiological link, CSF pleocytosis (> 5 cells/dL), and demonstration of recent TBEV infection by the presence of specific serum IgM and IgG antibodies (Kaiser 2008). After 2 weeks of hospitalization, patients underwent second lumbar puncture (control) for assessment of inflammatory features in CNS remission.

All patients were local inhabitants and did not report recent journeys abroad; therefore, there was no need for cross-reactive reaction with other *Flaviviridae* exclusion. To date, no confirmed human case of West Nile virus or yellow fever virus infection has been reported in Poland. None of the patients were vaccinated against TBE.

Sequelae were also analysed and classified as follows:

- Early subjective (< 1 month), such as headache and vertigo
- Early mental (< 1 month), such as memory impairment, loss of concentration, sleep disturbances, depression and anxiety
- Early neurological conditions (< 1 month), such as upper/lower limb paresis, cranial nerve paresis and cerebellar syndrome
- Late subjective (≥ 1 month), such as headache and vertigo
- Late mental factors (≥ 1 month), such as memory impairment, loss of concentration, sleep disturbances, depression and anxiety
- Late neurological conditions (≥ 1 month), such as upper/lower limb paresis, cranial nerve paresis, and cerebellar syndrome

The results are expressed as percentages, mean ± SD, medians, and ranges. Comparisons among groups were carried out using the chi-square test for dichotomous data or the Mann-Whitney *U* test for nonparametric distribution data. Correlations were performed with the Spearman test. All statistics were performed using STATSOFT STATISTICA, version 12 (Stat Soft, Inc., Tulsa, OK, USA).

The study was approved by the Local Bioethics Committee at the Medical University of Bialystok, Poland.

## Results

### General results

#### Group I

**Fig. 1:**
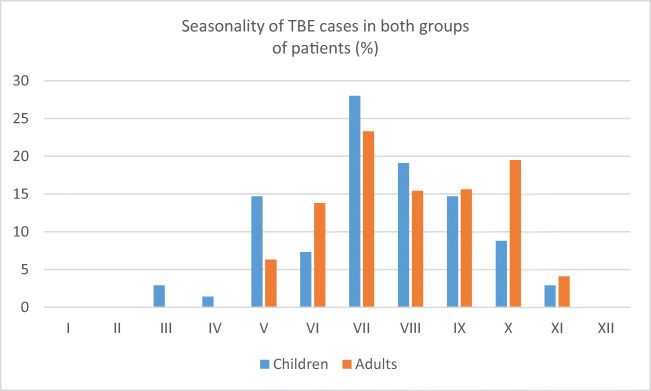
Seasonality of TBE cases in both groups of patients

Group I consisted of 31 (45.5%) females and 37 males (54.5%). A total of 43 (63%) patients were inhabitants of rural areas, and 25 (37%) lived in urban areas. Most of the cases were registered in May, July, August and September (Fig. 1). The duration of hospitalization was 18.05 ± 7.05 days. Thirty-four (45.5%) patients remembered a tick bite. The time period between tick bite and first symptom appearance was 22.3 ± 12.94 days. The time of fever duration at home was 4.2 ± 2.2 days. The time of home treatment was 4.23 ± 3.05 days. In 25 (36.7%) patients, the disease took a biphasic course.

#### Group II

Group II consisted of 230 (38.27%) females and 371 (61.73%) males. Two hundred eighty-five (47.42%) patients were inhabitants of villages, and 316 (52.58%) were inhabitants of cities. Most cases were registered in August, September, October and November (Fig. 1). The duration of the first hospitalization was 17.36 ± 5.39 days. Four hundred twenty-four (70.5%) patients remembered a tick bite. The time period between tick bite and first symptom appearance was 14.87 ± 11.9 days. The time of fever duration at home was 5.12 ± 4.58 days. The time of home treatment was 10.44 ± 7.28 days. In 198 (32.9%) patients, the disease took a biphasic course.

### Comparison of the symptoms in children and adults

A comparison of the symptoms in children and adults is presented in Table 1. In children, TBE usually takes the meningitis form, while in adults, meningoencephalitis and meningoencephalomyelitis are more often observed. In both groups, the dominant symptoms were headache and fever, but children more often complained about nausea and vomiting, while adults complained about vertigo. Serious neurological symptoms, such as limb/cranial nerve paresis, cerebellar syndrome and consciousness disturbances, were more common in adults.

**Table 1:** Comparison of the symptoms between children and adults

Symptom	Children		Adults		
	*n*	%	*n*	%	*p*
Course of the disease					
Meningitis	66	97	305	50.74	< 0.05
Meningoencephalitis	2	3	246	40.9	< 0.05
Meningoencephalomyelitis	1	1.5	50	8.3	< 0.05
Fatal outcome	0	0	7	1.16	< 0.05
Complaints					
Fever	63	92.64	542	90.2	NS
Mean fever	38.39 ± 0.945	–	38.9 ± 0.8	–	NS
Headache	66	97	568	94.5	NS
Vertigo	6	8.8	222	36.9	< 0.05
Nausea	59	86.76	307	51	< 0.05
Vomiting	48	70.59	219	36.3	< 0.05
Muscle pain	3	4.41	103	17.13	< 0.05
Objective symptoms					
Neck stiffness	59	86.76	470	78.2	< 0.05
Oppenheim’s sign	1	1.5	142	23.62	< 0.05
Babiński’s sign	1	1.5	43	7.14	< 0.05
Paresis—limbs	1	1.5	79	13.14	< 0.05
Paresis—cranial nerves	1	1.5	60	9.98	< 0.05
Cerebellar disturbances	2	3	131	21.8	< 0.05
Tremor	3	4.5	28	4.66	< 0.05
Speech disorders	4	5.8	25	4.15	NS
Sensation disorders	3	4.41	33	5.5	NS
Consciousness disorders	3	4.41	182	30.28	< 0.05
Bradycardia	1	1.5	20	3.32	< 0.05

### Comparison of the laboratory tests between children and adults

In group I, the C-reactive protein (CRP) concentration was elevated in 55 (80.9%) children and in 295 adults (49.1%) (*p* < 0.05), and white blood count (WBC) was elevated in 40 children (58.8%) and in 295 adults (49.1%) (*p* = 0.12). The erythrocyte sedimentation rate (ESR) was elevated in 50 children (75%) and in 275 (48.8%) adults (*p* < 0.05). Platelet (PLT) count was decreased in 8 children (11.8%) and 87 adults (14.5%) (*p* = 0.54), alanine aminotransferase (AlAT) activity was increased in 5 children (7.4%) and in 60 adults (10%) (*p* = 0.49), and aspartate aminotransferase (AspAT) activity was increased in 4 children (5.9%) and in 35 (5.8%) adults (*p* = 0.98). Cerebrospinal fluid (CSF) cytosis was increased in all children and adults. Protein concentration in CSF was increased in 25 children (36.8%) and 455 (75.7%) adults (*p* < 0.05).

A comparison of the results of laboratory tests between children and adults is presented in Table 2. In children, ESR and WBC at admission were higher than those in adults, and in adults, CRP concentration and PLT concentrations at admission were higher than those in children. CSF pleocytosis at admission was not significantly different between the groups, but the protein concentration was significantly higher in adults. In the second examination of CSF, there were significant differences in pleocytosis and protein concentration between children and adults.

**Table 2:** Comparison of the results of laboratory tests between children and adults

	Children					Adults					
	Mean	Median	Min	Max	SD	Mean	Median	Min	Max	SD	*p*
Age	12.16	14	2.5	18	4.54	45.90	47	17	82	16.49	-
ESR (1 h)	33.42	34.00	4	90	19.2	27.35	24	2	101	19.02	< 0.05
CRP (mg/dL)	22.59	14.45	0.1	141	36.45	25.34	10.4	0.2	203.7	34.31	< 0.05
WBC (× 10^3^/ml)	13,682.24	12,800	4480	28,200	5057.39	10,229.61	10,062.5	1330	38,900	3343.42	< 0.05
AspAT (U/L)	24.42	18	10	193	26.2	23.35	19	6	300	19.15	NS
AlAT (U/L)	19.87	14	4	185	24.56	24.81	19	4	418	26.57	< 0.05
PLT (× 10^9^/L)	263.9	250	133	470	77.31	284.6	270.5	119	503	94.46	< 0.05
CSF I–cytosis (cells/μl)	128.63	76.5	10	896	141.8	110.27	80	11	1268	112.32	NS
CSF I–protein (mg/ml)	52.42	39	0.9	172	36.18	65.24	60.85	16	265.5	25.89	< 0.05
CSF II–cytosis (cells/μl)	35.44	19	4	164	38.021	44.49	36	1	193	31.31	< 0.05
CSF II–protein (mg/ml)	39.87	29.45	10	105	25.634	64.57	56.4	14.9	221	33.84	< 0.05

### Dexamethasone treatment

In both groups, the treatment was symptomatic with analgesics, anti-inflammatory drugs and 20% mannitol. In patients with a hyperergic immunological response (consciousness disturbances, fever over 40 °C, persistent vomiting, brain oedema), dexamethasone was used.

In group I, 10 (14.7%) cases were treated with dexamethasone at a dosage of 1–16 mg/day for 1–11 days. In group II, 278 (46.2%) patients received dexamethasone at a dosage of 16–24 mg/day for 3–17 days.

The children who received dexamethasone over 7 days had a significantly higher pleocytosis II to pleocytosis I ratio (0.56 ± 0.289) than other patients (*p* < 0.05). Treatment with dexamethasone correlated with pleocytosis in the control CSF examination in children, which may indicate that dexamethasone usage prolongs the disease.

### Outcome of disease

Seven (10.29%) children had third lumbar puncture, while 175 (29.1%) adults required third lumbar puncture, and 4 (0.66%) even required a fifth lumbar puncture, which reflects the prolongation of the inflammatory process in adults.

The follow-up examination regarding sequelae analysis was performed in 22 children and 600 adults. Six (27%) children developed sequelae, while 253 (42.1%) adults developed sequelae.

Comparison of sequelae groups showed significant differences between all groups (*p* < 0.05) except early subjective (*p* = 0.2). There are differences in the frequency of all sequelae: headache, vertigo, memory impairment, loss of concentration, sleep disturbances, depression, anxiety, upper/lower limb paresis, cranial nerve paresis and cerebellar syndrome between children and adults.

There were no differences in sequelae development depending on the clinical course of the disease. There were no differences in sequelae development between children who received dexamethasone and those who did not.

### Co-infection with *Borrelia burgdorferi*

In children, 10 (14.8%) patients were co-infected with *Borrelia burgdorferi*. In adults, 118 (19.6%) patients were co-infected with *Borrelia burgdorferi*.

### Analysis of correlation

In group I, the duration of dexamethasone treatment correlated with pleocytosis in the control CSF examination. Pleocytosis at admission correlated with protein concentration in CSF and then with pleocytosis and protein concentration in the control CSF examination.

In group II, the duration of dexamethasone supply and initial dose of dexamethasone correlated with pleocytosis in the control CSF examination. ESR and CRP correlated with AlAT, AspAT activities, pleocytosis and protein concentration in the CSF at admission.

## Discussion

Our study is the first comparison of children and adults with TBE in Poland. The majority of the results of our study confirms the results obtained by authors from other countries. According to epidemiological data, the incidence of TBE in children is ten times lower than in adults (Kaiser 2016). The results of our study confirm this observation.

The incubation period of TBE usually varies from 4 to 28 days (Zajkowska et al. 2011) (on average approximately 10 days) (Logar et al. 2000). In our study, the incubation period was significantly longer in children (22.3 ± 12.94 days) than in adults (14.87 ± 11.9 days).

A total of 45.5% of children remembered a tick bite, similar to the results shown by Lesnicar et al. (47.9%) (Lesnicar et al. 2003).

TBE in children has a milder course than in adults. The most common clinical form of the disease is meningitis. In children, non-specific symptoms such as fever, headache, fatigue and exhaustion appear more frequently (Arnez and Avsic-Zupanc 2009). In the study by Lesnicar et al., the most common symptoms and signs of TBE included fever (> 38 °C (100% of patients)), headache and meningeal signs (93%), fatigue (91%) and vomiting (88%) (Lesnicar et al. 2003). In our study, children most frequently complained of fever, headache, nausea and vomiting.

Many studies report that the biphasic course is one of the hallmark symptoms of TBE. Our study showed that only 36.7% of children and 32.9% of adults presented with the biphasic course.

The disease caused by European TBEV generally has a milder course and better outcome in children than in adults. Nevertheless, TBE cases with a severe clinical course, permanent sequelae and even death have been described, not only in adults but also in children (Fowler et al. 2013). Children may experience long-term disturbances of attention and concentration, but the available data vary (Arnez and Avsic-Zupanc 2009). Our study confirms these observations, as the majority of the children developed mild lymphocytic meningitis, while almost 50% of adults developed a severe form of meningoencephalitis or meningoencephalomyelitis. We did not observe any fatal outcome of the disease in children, while in adults, 7 patients out of 601 died. Based on a summary of 8 studies on 1169 children with TBE from 1963 to 2005, Arnez et al. showed that meningitis was present in 802 (69%) patients, meningoencephalitis in 356 (30%) patients and meningoencephalomyelitis in 11 (1%) patients (Arnez and Avsic-Zupanc 2009).

Diagnosis of TBE is based on CSF examination, where lymphocytic pleocytosis and increased protein concentration are observed, and positive results of serological tests for TBEV. In our study, significant differences in protein concentration in the CSF examination (at admission and in control examination) between adults and children were observed. Additionally, differences in pleocytosis in the control examination but not in pleocytosis at admission were stated. It may be indirect proof that TBE affects the brain more frequently in adults than in children.

The treatment of TBE is symptomatic, but when the disease takes a severe course, dexamethasone may be used. In our study, some adults as well as children received dexamethasone. We observed that treatment with dexamethasone correlated positively with pleocytosis in the checkup CSF in children. There were no significant differences in sequelae development between children treated and not treated with dexamethasone. This may indicate that dexamethasone usage prolongs the disease but does not influence sequelae development. However, since the treatment with dexamethasone is more a causal result than a controlled study, the finding should be interpreted with caution.

The World Health Organization (WHO) recommends vaccination for people of all age groups, including children, in highly endemic areas (≥ 5 cases/100,000 per year). The disease in children is generally milder, although severe illness may occur and even lead to permanent impairment of the quality of life owing to neuropsychological sequelae. Therefore, immunization should be offered to all children living in or travelling to endemic areas (Kunze et al. 2004).

## Conclusions

1. The presentation of TBE in children is milder than in adults.
2. Nausea and vomiting are more frequent in children, while neurological manifestations are more frequent in adults.
3. There were no differences in cerebrospinal fluid pleocytosis between children and adults with TBE, whereas the protein concentration was higher in adults on admission.
4. Sequelae after TBE are less frequent in children than in adults.
5. Dexamethasone usage prolongs the disease but does not influence sequelae development.
